# Post-traumatic and dissociative depression in a sample of Chinese high school students

**DOI:** 10.1192/j.eurpsy.2026.11882

**Published:** 2026-07-31

**Authors:** C. T. L. Chan, S. K. K. Lam, G. F. Yuan, A. K. C. Chau, H. W. Fung

**Affiliations:** 1 School of Nursing, The Hong Kong Polytechnic University; 2 School of Nursing and Health Sciences, Hong Kong Metropolitan University, Hong Kong, Hong Kong; 3 School of Education Science, Leshan Normal University, Leshan, China; 4 Department of Psychiatry, The University of Hong Kong, Hong Kong, Hong Kong

## Abstract

**Introduction:**

Depression commonly co-occurs with trauma-related conditions, such as post-traumatic stress disorder (PTSD), but less is known about this comorbidity among adolescents, especially since the launch of ICD-11. Moreover, dissociation is also a trauma-related symptom that may be common in people with depression. Very few studies looked into PTSD and dissociation in adolescents with and without probable depression, although there may be a post-traumatic or dissociative subtype of depression.

**Objectives:**

This study investigated ICD-11 PTSD, complex PTSD (CPTSD), and dissociation among Chinese high school students who screened positive for depression. We explored differences in childhood maltreatment, household dysfunctions, and psychotic symptoms in depressed participants with and without PTSD/dissociation.

**Methods:**

We recruited high school students in two public schools in China to complete the PHQ-9, the 10-item Adverse Childhood Experiences Questionnaire, the International Trauma Questionnaire, the Dissociative Experiences Scale-Taxon (DES-T), and the Community Assessment of Psychic Experiences–Positive (CAPE-P15).

**Results:**

In total, 1,147 high school students (mean age = 16.40; 54.6% female) completed the assessments; 204 (11.78%) screened positive for depression (PHQ-9 ≥ 10). Of students with depression, 4.4% screened positive for PTSD, 24.5% screened positive for CPTSD; 46.6% screened positive for dissociation (DES-T ≥ 28). In total, of students with depression, 55.9% had either or both PTSD/CPTSD and dissociation. Depressed students with PTSD/dissociation (n = 114) reported significantly more childhood maltreatment (M = 1.52; SD = 1.47 vs 0.97; SD = 1.26)(t = 2.81, p = .004), more household dysfunctions (M = 0.78; SD = 1.25 vs M = 0.42; SD = 0.81)(t = 2.492, p = .014), and more positive symptoms of psychosis (M = 1.31; SD = 0.69 vs M = 0.65; SD = 0.49)(t = 7.959, p < .001), compared with depressed students without PTSD/dissociation (n = 90).

**Conclusions:**

Over half (55.9%) of depressed students in this study also experienced trauma-related symptoms. These students reported significantly higher levels of childhood maltreatment, household dysfunctions, and positive symptoms of psychosis compared to those with only depression. These findings emphasize the importance of thorough mental health evaluations and targeted interventions to address trauma and dissociation in depressed adolescents.

**Disclosure of Interest:**

None Declared

